# Resonant-Based Wireless Power Transfer System Using Electric Coupling for Transparent Wearable Devices and Null Power Points

**DOI:** 10.3390/s23031535

**Published:** 2023-01-30

**Authors:** Kyeungwon Bang, Hongguk Bae, Sangwook Park

**Affiliations:** Department of Electronic Engineering, Daegu University, Gyeongsan 38453, Republic of Korea

**Keywords:** electric coupling, resonant-based WPT, null power point, metal mesh

## Abstract

**Simple Summary:**

Wireless power transfer using electric coupling can deliver the power by electric field between two metal plates, and a copper plate is generally used for metal material. This study presents that a transparent and flexible metal mesh material can replace the existing copper plate in resonance-based wireless power transfer using electric coupling. In wireless power transfer using electric coupling, a phenomenon called null power point occurs depending on the relative positions of the transmitter and receiver, which negatively affects the efficiency of power transfer. This study also clarified the reason for the occurrence of null power points in the power transfer process.

**Abstract:**

This study provides information on the transfer efficiency of four-plate-structured copper plate and metal mesh sheet couplers, the cause of null-power point. The couplers are compared based on the equivalent circuit model analysis, experimental results of fabricated couplers, and simulation results of the High-Frequency Structure Simulator (HFSS) tool. It was confirmed that the metal mesh material exhibits the same performance as the existing copper plate and can be fully used as a coupler material for the electrical resonance wireless power transfer system. In addition, the null-power point phenomenon is only determined by the main coupling and cross coupling between the transmitter and receiver, which are most dominantly affected by the coupler structure.

## 1. Introduction

The electrical resonance wireless power transfer (ER-WPT) method transmits power without any direct contact based on alternating electric field coupling between two couplers. The coupler of the magnetic resonance wireless power transfer (MR-WPT) system based on magnetic field coupling incurs a high cost for the system and heavy weight because of a mass of litz wires and ferrite plates. There are other disadvantages, e.g., power loss in coil winding, eddy current loss in the nearby medium, and adverse effects on humans owing to electromagnetic interference [1,2]. In the ER-WPT system, on the other hand, a metal plate is usually used, which makes a lighter coupler than coil and can be implemented at a low cost. In addition, the electric field in ER-WPT can pass through the metal plate without significant power loss [2,3]. The closer the transfer distance, the greater the reduction in the fringing effect, and the electric field is only limited between the metal plates; therefore, it can be safely used regardless of the surrounding environment [4].

ER-WPT is evaluated as an excellent alternative to the MR-WPT method. It is widely used in short-range applications (mW to kW levels), that is, low-power biomedical devices, charging drones, and high-power electric vehicles [5,6,7]. In recent times, to reduce the inductance required for resonance, a high-frequency (MHz or higher) AC voltage source has been used and included a matching network and rectifier structure. The coupler of the ER-WPT system mainly consists of copper and aluminum plates due to the high conductivity and low cost. However, to achieve high-efficiency WPT despite the shape change of the coupler, there has been a recent study of grafting a conductor with high flexibility and transparency. This study has been conducted to improve user convenience in wearable applications [8,9]. However, these studies were conducted using the MR-WPT method only. There is a study that uses graphene film material to ER-WPT for wearable biomedical sensors [10], but the graphene film material is quite different from the metal mesh film material, and the system only implements series resonance without matching. In a recent study about the four-plate coupler ER-WPT system, the coupling capacitance component in the misalignment state was analyzed [11]. However, it only shows the change in each capacitor component in the coupler, with no discussion about the null power point phenomenon, which can occur in the four-plate coupler. The contribution of this study can be summarized in two parts.

1. Metal mesh (flexible and transparent material) is used as an ER-WPT coupler and its performance is compared with conventional copper plate material.
2. A null power point analysis is conducted and clarifies the reason why the null power point occurs in ER-WPT.

In this study, a four-plate coupler structure is implemented using metal mesh and copper plate materials, and the characteristics of both materials are measured and compared. An analysis of the null power point phenomenon during misalignment is also evaluated using the $S_{21}$ parameter. Based on actual measurements, it is concluded that a coupler using a metal mesh material can exhibit almost the same characteristics and performance as a copper plate and be used as an ER-WPT coupler material instead of the copper plate. The metal mesh material is more flexible than the copper plate; thus, the shape and structure of the coupler can be designed to be more flexible and lightweight.

## 2. WPT System Model

### 2.1. Configuration of ER-WPT System

**Figure 1:**
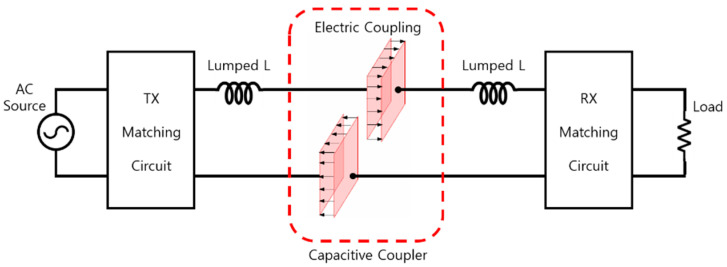
Block diagram of proposed ER-WPT system.

Figure 1 shows the block diagram of the ER-WPT system to which the proposed capacitive coupler is applied. The AC voltage source generates AC voltage with a desired operating frequency, and the proposed system is designed to possess a resonance frequency of 6.78 MHz. The generated current passes through a matching circuit that matches the impedance of primary and secondary side, and the Lumped-L, which will generate resonance at the desired frequency, and is delivered to the plate of the coupler. The coupler includes a four-plate structure, and the electrical coupling occurring between the plates facing each other operates as a capacitive element. Power can be wirelessly transferred from the primary to the secondary through the electrical coupling.

### 2.2. Structure of Capacitive Coupler

**Figure 2:**
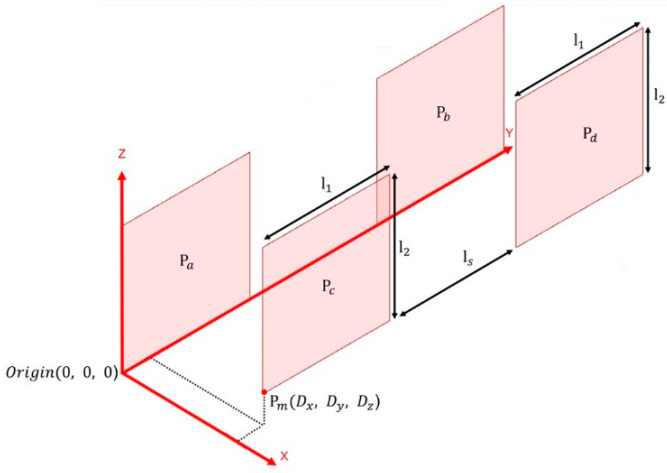
Parameters of the proposed capacitive coupler.

The four-plate coupler structure shown in Figure 2 is used in the ER-WPT system [12,13,14] and comprises four metal plates, i.e., $P_{a},\;P_{b},\;P_{c}$, and $P_{d}$. The horizontal and vertical lengths are $l_{1}$ and $l_{2}$, respectively, and $l_{s}$ between the two plates is used. $l_{1}$ and $l_{2}$ of the proposed coupler each measure 100 mm, and $l_{s}$ is also designed to be 100 mm.

The $P_{a}$ and $P_{b}$ plates are defined as the primary side, and the $P_{c}$ and $P_{d}$ plates are arranged as the secondary side. In general, the secondary side is separated from the primary side by $D_{x}=10\;\mathrm{mm}$, and in a misalignment situation, it can be moved along the $D_{y}$ and $D_{z}$ directions (denoted as a misaligned point $P_{m}$).

**Figure 3:**
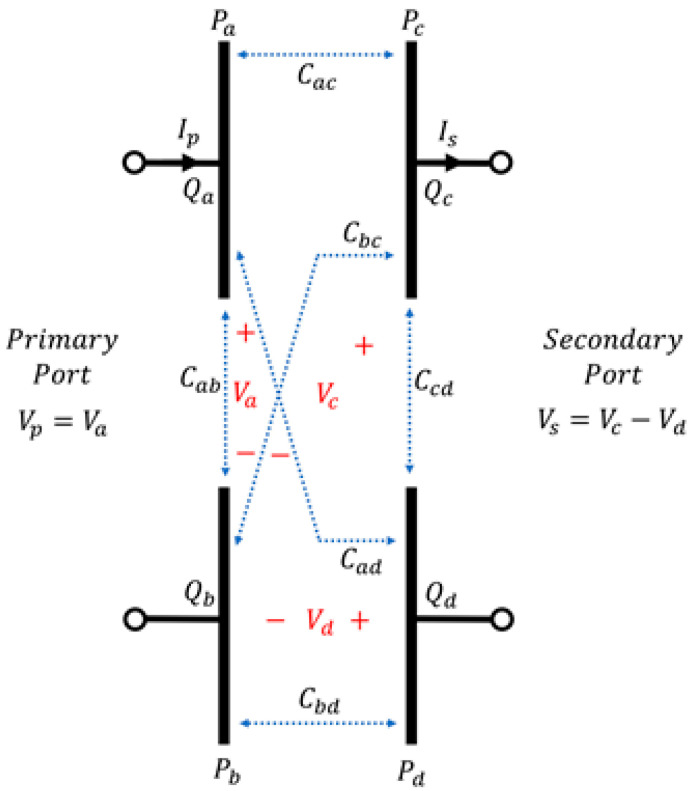
Physical coupler model.

**Figure 4:**
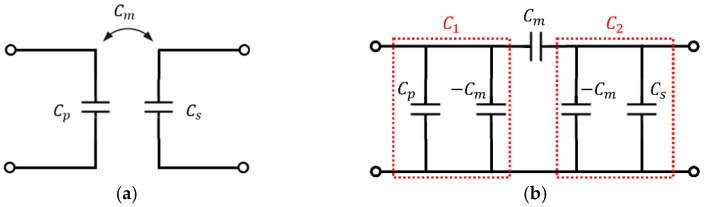
Circuit model of the capacitive coupler. (**a**) Basic illustration of the capacitive coupler. (**b**) Pi model.

As seen from Figure 3, the four-plate coupler structure includes six coupling capacitances. $C_{ac}$ and $C_{bd}$ are formed by two facing plates known as main-coupling capacitances. The capacitances formed by the two plates on the same side are self-capacitances (denoted as $C_{ab}$ and $C_{cd}$). The capacitances in the opposite direction are known as cross-coupling capacitances (denoted as $C_{bc}$ and $C_{ad}$). The equivalent circuit of the four-plate coupler structure can be presented, as shown in Figure 4a, with the mutual capacitance $C_{m}$ and the equivalent capacitances $C_{p}$ and $C_{s}$ on both sides. If $V_{p}$ and $V_{s}$ are the voltage that defines the primary port and secondary port voltage, the electric charge of each plate is $Q_{a}$, $Q_{b},\;Q_{c}$, and $Q_{d}$; the voltage is defined as $V_{a}$, $V_{b},\;V_{c}$, and $V_{d}$. If $V_{b}$ is set as the reference voltage, i.e., $V_{b}=0$, the primary input voltage can be defined as $V_{p}$= $V_{a}$, and the secondary output voltage can be defined as $V_{s}=V_{c}-V_{d}$. If Kirchhoff’s current law is applied, the relationship between the voltage and current of each plate can be expressed as a matrix, as defined in Equation (1). The self-capacitance components $C_{aa},\;C_{bb},C_{cc}$, and $C_{dd}$ are not considered because they are smaller than the capacitance between any two plates [15].

(1) $$\left[\begin{array}{c} \begin{array}{c} Q_{a}\\ Q_{b} \end{array}\\ Q_{c}\\ Q_{d} \end{array}\right]=\left[\begin{array}{c} \begin{array}{c} I_{p}/j\omega \\ -I_{p}/j\omega \end{array}\\ -I_{s}/j\omega \\ I_{s}/j\omega \end{array}\right]=\left[\begin{array}{cccc} C_{a} & -C_{\mathrm{ab}} & -C_{\mathrm{ac}} & -C_{\mathrm{ad}}\\ -C_{\mathrm{ab}} & C_{b} & -C_{\mathrm{bc}} & -C_{\mathrm{bd}}\\ -C_{\mathrm{ac}} & -C_{\mathrm{bc}} & C_{c} & -C_{\mathrm{cd}}\\ -C_{\mathrm{ad}} & -C_{\mathrm{bd}} & -C_{\mathrm{cd}} & C_{d} \end{array}\right]\left[\begin{array}{c} V_{a}\\ 0\\ V_{c}\\ V_{d} \end{array}\right]$$

where

$$\left\{\begin{array}{c} C_{a}=C_{aa}+C_{ab}+C_{ac}+C_{ad}\approx C_{ab}+C_{ac}+C_{ad}\\ C_{b}=C_{ab}+C_{bb}+C_{bc}+C_{bd}\approx C_{ab}+C_{bc}+C_{bd}\\ C_{c}=C_{ac}+C_{bc}+C_{cc}+C_{cd}\approx C_{ac}+C_{bc}+C_{cd}\\ C_{d}=C_{ad}+C_{bd}+C_{cd}+C_{dd}\approx C_{ad}+C_{bd}+C_{cd} \end{array}\right.$$

The matrix of Equation (1) and the relationship among $V_{p},\;I_{p}$, and $V_{s},I_{s}$ can be expressed as (2) and (3)

(2) $$\begin{array}{ll} & V_{p}=\frac{I_{p}}{j\omega }\cdot \frac{1}{\left[C_{ab}+\frac{\left(C_{ac}+C_{ad}\right)\left(C_{bc}+C_{bd}\right)}{\left(C_{ac}+C_{ad}+C_{bc}+C_{bd}\right)}\right]}\\ & +\frac{V_{s}\left(C_{ac}\;C_{bd}-C_{ad}\;C_{bc}\right)}{C_{ab}\left(C_{ac}+C_{ad}+C_{bc}+C_{bd}\right)+\left(C_{ac}+C_{ad}\right)\left(C_{bc}+C_{bd}\right)} \end{array}$$

Using a similar method, the relationship among $V_{s}$, $I_{s}$, and $V_{p}$ can be expressed as

(3) $$\begin{array}{ll} & V_{s}=\frac{I_{s}}{j\omega }\cdot \frac{1}{\left[C_{cd}+\frac{\left(C_{ac}+C_{bc}\right)\left(C_{ad}+C_{bd}\right)}{\left(C_{ac}+C_{ad}+C_{bc}+C_{bd}\right)}\right]}\\ & +\frac{V_{p}\left(C_{ac}\;C_{bd}-C_{ad}\;C_{bc}\right)}{C_{cd}\left(C_{ac}+C_{ad}+C_{bc}+C_{bd}\right)+\left(C_{ac}+C_{bc}\right)\left(C_{ad}+C_{bd}\right)} \end{array}$$

Equations (2) and (3) are combined to obtain equivalent capacitances $C_{p}$, $C_{s}$, and $C_{m}$ on the primary and secondary sides, as shown in Figure 4a.

(4) $$\left\{\begin{array}{c} C_{p}=C_{ab}+\frac{\left(C_{ac}+C_{ad}\right)\left(C_{bc}+C_{bd}\right)}{\left(C_{ac}+C_{ad}+C_{bc}+C_{bd}\right)}\\ C_{s}=C_{cd}+\frac{\left(C_{ac}+C_{bc}\right)\left(C_{ad}+C_{bd}\right)}{\left(C_{ac}+C_{ad}+C_{bc}+C_{bd}\right)}\\ C_{m}=\frac{C_{ac}\;C_{bd}-C_{ad}\;C_{bc}}{\left(C_{ac}+C_{ad}+C_{bc}+C_{bd}\right)} \end{array}\right.$$

Using the above method, the four-plate coupler can be modeled as shown in Figure 4a. In addition, the Pi model equivalent circuit is used to represent two capacitors sharing an electric field in Figure 4b. The equivalent capacitances, $C_{1}$ and $C_{2}$, can be obtained using Equation (4) as follows.

(5) $$C_{1}=C_{p}-C_{m}=C_{ab}+\frac{C_{ac}C_{bc}+C_{ad}C_{bd}+2\left(C_{ad}C_{bc}\right)}{\left(C_{ac}+C_{ad}+C_{bc}+C_{bd}\right)}$$

(6) $$C_{2}=C_{s}-C_{m}=C_{cd}+\frac{C_{ac}C_{ad}+C_{bc}C_{bd}+2\left(C_{ad}C_{bc}\right)}{\left(C_{ac}+C_{ad}+C_{bc}+C_{bd}\right)}$$

### 2.3. Measured Equivalent Circuit Model and Power Efficiency

**Figure 5:**
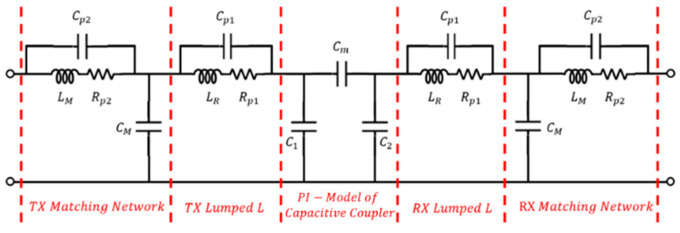
Practical equivalent circuit model.

Figure 5 shows the practical equivalent circuit of the ER-WPT system of this study including the Pi model. For resonance in several MHz bands, the parasitic components of the capacitor are not considered because they are negligibly small; however, the parasitic component of an inductor is non-negligible and every parasitic is considered. To define the capacitance based on the actual measured values, six capacitance components between each plate shown in Figure 3 were measured using an RLC meter. The measurement was conducted on the copper plate and metal mesh materials at $D_{x}=10\;\mathrm{mm}$, and the calculated $C_{1},C_{2}$, and $C_{m}$ values are listed in Table 1.

As a result of the measurement, the copper plate and metal mesh exhibit almost same capacitances under the same $D_{x}$. LCL matching was applied to the fabricated system [6]. The value of the components used in the circuit are listed in Table 2.

The large external inductor $L_{R}$ in the transmitter and receiver side is connected in series with a capacitive coupler to decrease the resonant frequency of the system. The combination of $L_{M}$ and $C_{M}$ operates like low-pass filter. Therefore, the high-order harmonics components cannot flow into the coupler.

**Figure 6:**
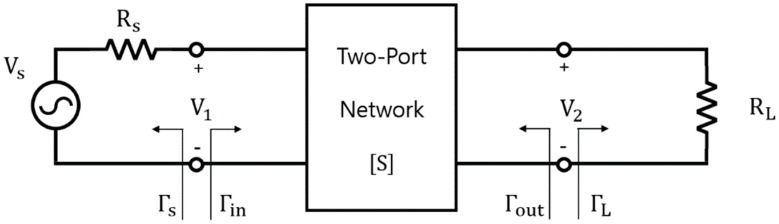
Block diagram of two-port network [16].

A general two-port network is shown in Figure 6, and the WPT efficiency $\eta =P_{L}/P_{in}$ can be expressed as in [16].

(7) $$\eta =\frac{P_{L}}{P_{in}}=\frac{{\left|S_{21}\right|}^{2}\left(1-{\left|\Gamma_{L}\right|}^{2}\right)}{\left(1-{\left|\Gamma_{in}\right|}^{2}\right){\left|1-S_{22}\Gamma_{L}\right|}^{2}}$$

where:

$P_{in}$: average power supplied to the network.

$P_{L}$: average power supplied to the load.

$\Gamma_{s}$: reflection coefficient seen looking toward the source.

$\Gamma_{in}$: reflection coefficient seen looking into Port 2 of the network where Port 2 is terminated by $Z_{L}$.

$\Gamma_{out}$: reflection coefficient seen looking into port 2 of the network when port 1 is terminated by $Z_{S}$.

$\Gamma_{L}$: reflection coefficient seen looking toward the load.

As the reflection coefficients $\Gamma_{in}$ and $\Gamma_{L}$ are 0 under the condition that the circuit is perfectly resonant, ${\left|S_{21}\right|}^{2}$ implies the same concept as the efficiency of WPT.

### 2.4. Null-Power Point and Mutual Capacitance

Since, in mutual capacitive coupling, the higher the better, research has been conducted to increase the mutual capacitance [17]. The null power point is a phenomenon in which transfer characteristics are temporarily lost in a specific misalignment point [18]. It occurs when the $C_{m}$ becomes zero, and the reason $C_{m}$ becomes zero in the misalignment situation is that the product $C_{Ma}$ of the two main-coupling capacitance components ($C_{ac}$ and $C_{bd}$) and the product $C_{Cro}$ of the cross-coupling capacitance components ($C_{bc}$ and $C_{ad}$) become the same as Equation (8).

(8) $$C_{ac}\;C_{bd}=C_{bc}\;C_{ad}\;↔\;C_{Ma}=C_{Cro}$$

Therefore, at the point where Equation (8) is satisfied, even though the coupler is located within the effective charging area, power transfer is impossible.

## 3. Results and Discussion

### 3.1. Verification of the Proposed Approach

#### 3.1.1. Fabricated Model

**Figure 7:**
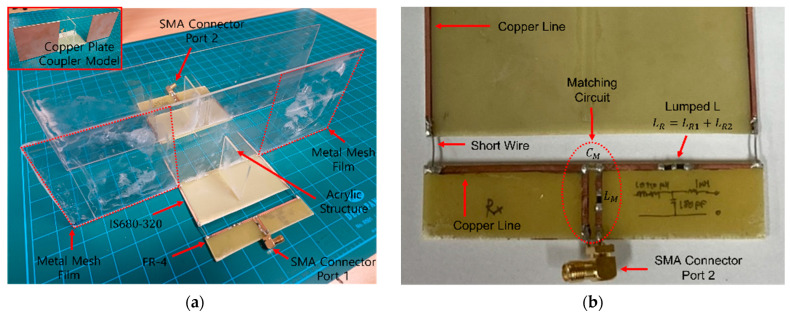
Fabricated proposed ER-WPT system. (**a**) Fabricated model. (**b**) Matching circuit structure.

Figure 7 shows the fabricated four-plate coupler structure using metal mesh and copper plate for the ER-WPT system. As shown in Figure 7a, a pair of metal mesh couplers is fabricated for the ER-WPT system, and a pair of copper plate couplers of the same size and structure is also fabricated, as shown at the top of Figure 7a. An acrylic structure is used to fix the plate at the desired position. In addition, to place the matching circuit far enough from the coupler, IS680-320 substrate is used, which has $\varepsilon_{r1}=3.2$ and all the conductors were removed except for the copper line. The IS680-320 board is connected to the FR-4 board that contains the Lumped-L and matching circuit through a short wire. The dielectric constant of the FR-4 substrate is $\varepsilon_{r2}=4.4$, and the parameters of the components listed in Table 2 are attached to the T-shaped copper line structure, as shown in Figure 7b. In the case of the $L_{R}$, the two inductors of $L_{R1}$ and $L_{R2}$ are connected in series to match the appropriate inductance. To minimize the parasitic components, the circuit and path should be simplified; however, the circuit structure is designed to improve the convenience of the measurements by replacing various matching circuits without significantly affecting the system. Finally, an SMA connector is used for vector network analyzer (VNA) measurements.

**Figure 8:**
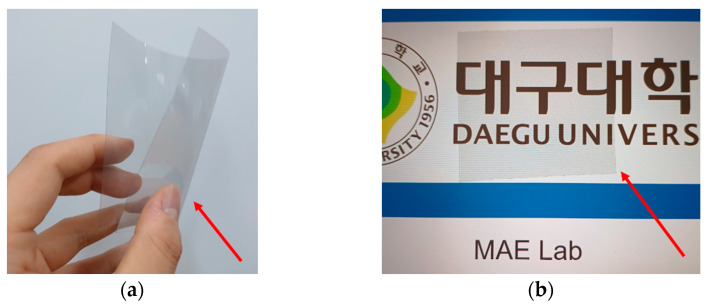
Metal mesh film characteristics. (**a**) Flexibility. (**b**) Transparency.

Figure 8 shows the metal mesh material. The metal mesh material is a transparent electrode made of a micro copper wire and a conductor with light transmittance and flexibility. The metal mesh used in this study has a light transmittance, thickness, and conductivity of 80%, 260 nm, and 1.51E + 06 S/m, respectively.

#### 3.1.2. Experimental and Simulation Results

The simulation using an electromagnetic analysis tool (HFSS) and equivalent circuit analysis program was conducted. The practical equivalent circuit model was used for equivalent circuit (EC) analysis in Figure 5, and all parameters listed in Table 2 and Table 3 were applied for each component. The analysis results were compared and verified with the VNA measurement results.

**Figure 9:**
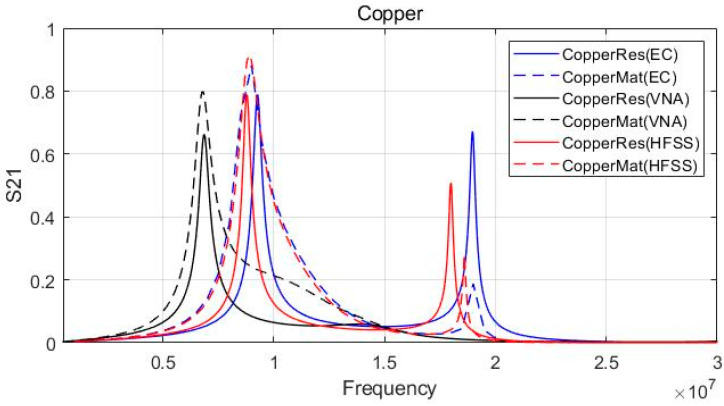
Experimental and simulation result (copper plate).

Figure 9 shows the equivalent circuit model analysis, the experimental results based on the VNA measurements, and the electromagnetic analysis simulation results at the same time. Each result is expressed as an S-Parameter at $D_{x}=10\;\mathrm{mm}$. Despite applying a matching circuit that resonates at 6.78 MHz, the equivalent circuit model and electromagnetic simulation results have a 9 MHz resonance frequency, unlike the VNA measurement result. The reason for the discrepancy between the simulation (EC, HFSS) and VNA results is that the parasitic capacitance stemming from fabrication influences the system as a self-capacitance component. Even though we considered the parasitic circuit components in Table 2, The additional parasitic components during fabrication also affect the completed model. Except for the resonance frequency mismatch with the VNA measurement results, all results show good agreement. The $S_{21}$ is improved after matching, and the bandwidth of the resonant frequency is increased.

**Figure 10:**
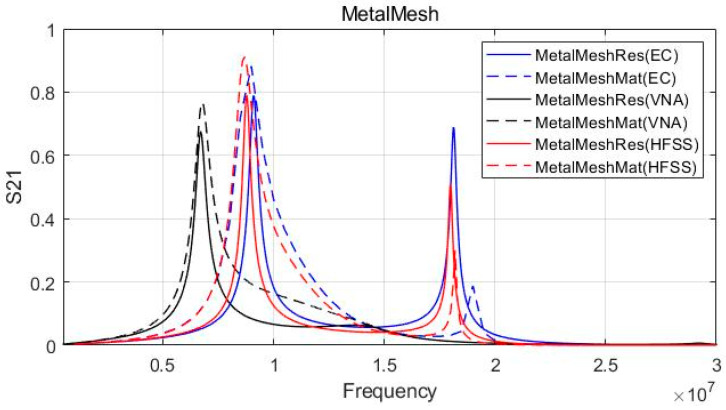
Experimental and simulation result (metal mesh).

Figure 10 shows the results of the metal mesh coupler at $D_{x}=10\;\mathrm{mm}$. The metal mesh demonstrates almost the same result as using a copper plate. This result validates Table 1 in that the $C_{1}$, $C_{2}$, and $C_{m}$ values of the copper plate and metal mesh are nearly identical. Thus, the subsequent simulation results only indicated the metal mesh coupler case. Except for the resonant frequency error of the VNA measurement, it was possible to verify that all three results exhibited similar trends and the magnitude of $S_{21}$. Therefore, the results obtained based on the simulation and equivalent circuit analysis can be regarded as reasonable.

### 3.2. Power Efficiency Calculated Based on the Proposed Approach

Since it was confirmed in the previous section that the HFSS simulation and equivalent circuit analysis results were reliable, the transmission characteristics of the coupler were analyzed at various distances and misalignment cases. To evaluate the transmission characteristics, the $C_{m}$ and ${\left|S_{21}\right|}^{2}$ were plotted.

**Figure 11:**
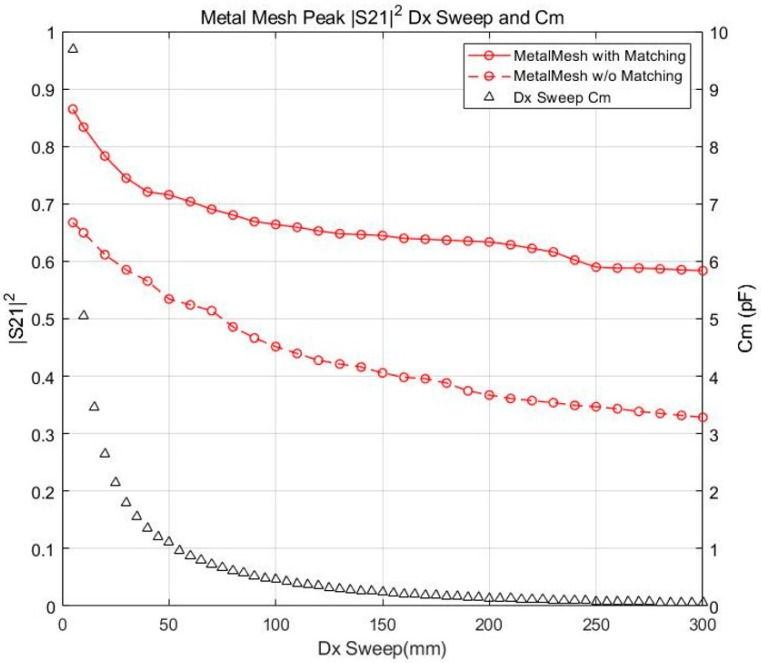
Efficiency and mutual capacitance variation under distance ($D_{x}$) sweep: 5–300 mm.

Figure 11 shows the maximum values of ${\left|S_{21}\right|}^{2}$ before and after matching based on the $D_{x}$ of the metal mesh coupler. $D_{x}$ swept from 5 to 300 mm. The efficiency of the metal mesh after matching significantly improved by 0.2–0.25 compared to without matching. $C_{m}$ had a high value of 10 pF at $D_{x}=5\;\mathrm{mm}$, but exponentially decreased to 1 pF when $D_{x}$ increased from 5 to 50 mm. As the $D_{x}$ increased beyond, it gradually converged to 0 pF. When $D_{x}$ changed from 10 mm to 50 mm, there was sharp decrease in efficiency both after and before matching, i.e., 0.87–0.71 and 0.68–0.54, respectively. When $D_{x}$ changed from 50 mm to 300 mm, the efficiency gradually decreased from 0.71 to 0.59 and 0.54 to 0.34 after and before matching, respectively. A similar trend for $C_{m}$ and ${\left|S_{21}\right|}^{2}$ was also observed.

**Figure 12:**
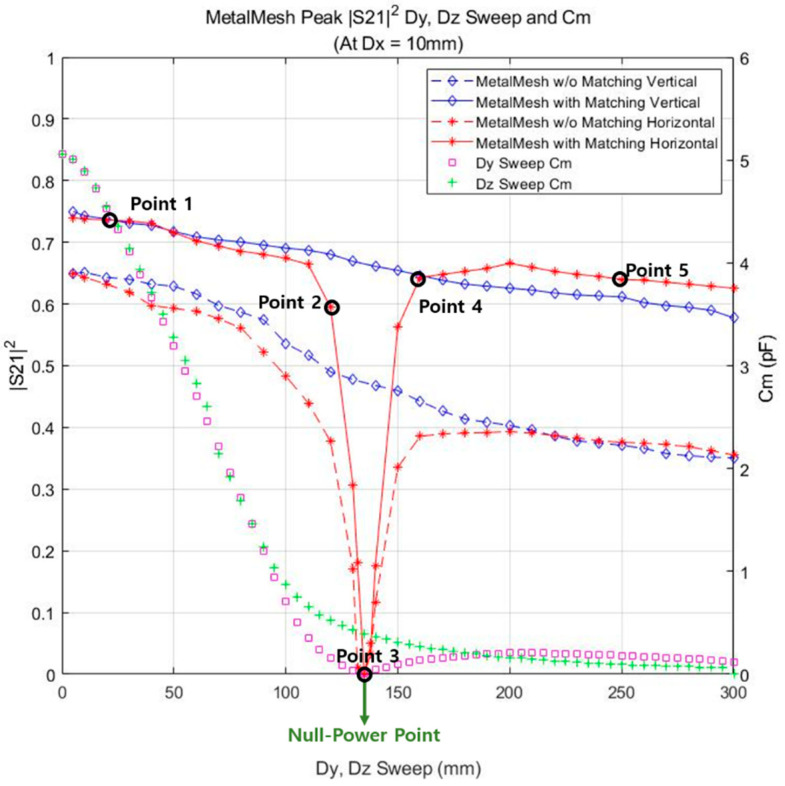
Efficiency and mutual capacitance variation under distance ($D_{y},D_{z}$) sweep: 0–300 mm.

Figure 12 shows the maximum values of ${\left|S_{21}\right|}^{2}$ before and after matching with 0 to 300 mm $D_{y}$ and $D_{z}$ misalignment case. $D_{x}$ is fixed at 10 mm in every case. In $D_{y}$ and $D_{z}$ misalignment situations, the efficiency after matching remarkably improved (0.1–0.25) compared to without matching. Both $C_{m}$ values for the change of $D_{y}$ and $D_{z}$ rapidly decreased from 5 to 0.8 pF together until $D_{y}$ and $D_{z}=100\;\mathrm{mm}$. After the $D_{z}$ exceeded 100 mm, the $C_{m}$ gradually converged to 0 pF until 300 mm. The efficiency before and after matching in the $D_{z}$ misalignment tended to uniformly decrease. Consequently, in the case of the $D_{z}$ misalignment, the efficiency and $C_{m}$ variation exhibited a similar decreasing trend with the $D_{x}$ sweep in Figure 11. After the $D_{y}$ exceeded 100 mm, the sharp decrease of $C_{m}$ was not alleviated, unlike the $C_{m}$ of the $D_{z}$ misalignment. Then, the $C_{m}$ value became 0 pF at $D_{y}=135\;\mathrm{mm}$ and slightly increased to 0.3 pF until 200 mm. The $C_{m}$ value of the $D_{y}$ misalignment demonstrated a clear difference from the $C_{m}$ of $D_{x}$ and $D_{z}$ in that it became zero at $D_{y}=135\;\mathrm{mm}$. Until $D_{y}=80\;\mathrm{mm}$, the efficiency before and after matching gradually decreased from 0.65 to 0.58 and 0.75 to 0.69, respectively. Although the after matching efficiency still gradually decreased in the range from 80 mm to 120 mm, the before matching efficiency started to decrease exponentially from 80 mm. In the end, both efficiencies simultaneously reached zero at $D_{y}$ = 135 mm. There was a noticeable increase in the efficiency between 135 and 160 mm, where it returned to the same level of efficiency as before, i.e., 0.39 and 0.65 for before and after matching, respectively. Both efficiencies slightly increased up to 200 mm and continued to decrease until 300 mm. As seen from this result, if only a minimum of $C_{m}$ exists, a moderate level of efficiency of 0.6–0.65 can be achieved with matching alone. However, in order to increase the transfer efficiency of the system above 0.65, the matching condition alone is not enough and the $C_{m}$ needs to be much larger; thus, the value of $D_{x}$ needs to be lower in a misalignment situation. These characteristics can be selected according to the user application as a trade-off.

### 3.3. Null Power Point

Depending on the misalignment direction, certain cross-capacitance components can rapidly increase, and during the main-coupling capacitance components can significantly decrease. From the specific misalignment point, the cross-coupling component becomes more dominant than the main-coupling component in determining $C_{m}$. This is known as the null power point and refers to the point where the coupling is not properly formed even if the receiver is within the effective charging area.

In the proposed ER-WPT system, the null power point can be observed at $D_{y}=135\;\mathrm{mm}$. Table 3 lists the main and cross-capacitor components and $C_{m}$ for five points around the null power point. $D_{y}=20,\;120,\;135,\;160,\;250\;\mathrm{mm}$ points were selected around 135 mm. The absolute values are taken to prevent $C_{m}$ from being negative. As seen from Table 3, the $C_{Ma}$ is larger than the $C_{Cro}$ at Point 1, which means main-coupling is much more dominant to compose $C_{m}$. At Point 2, the $C_{Ma}$ component is still larger than the $C_{Cro}$ component; however, the difference is not significant. The $C_{Ma}$ becomes equal to $C_{Cro}$ at Point 3, which can be defined as the null power point. After Point 3, the dominant component constituting $C_{m}$ is the $C_{Cro}$. At five points, cm is calculated as 4.528, 0.156, 0.006, 0.134, and 0.18 pF, respectively. In conclusion, the null power point does not occur unless the cross-capacitance component becomes larger than the main-coupling capacitance.

## 4. Conclusions

In this study, the four-plate structure consisting of copper plates and a metal mesh coupler for the ER-WPT system was implemented and measured. The validity was confirmed by comparing them with the HFSS simulation and the circuit analysis results using the Pi model. As a result, it was confirmed that the coupler using the metal mesh material exhibited the same performance as the copper plates. Therefore, the metal mesh material can be used as a substitute for the copper plate. It can be applied to various applications that cannot be realized with conventional copper plates, such as watchstraps, wearable biomedical devices, and curved electric devices. The analysis of the null power point was also conducted. The cause of the null power point is that the mutual capacitance becomes zero at the point where the product component of the cross-capacitances becomes larger than the product component of the coupling capacitances; it occurs in ER-WPT systems with multiple plates. This phenomenon is only determined by the structure of the coupler and is not related with any other factors, such as matching state or circuitry characteristics. In this study, the null power is found at $D_{y}=135\;\mathrm{mm}$; however, it may vary depending on the shape and design of the coupler. In order to overcome the null power point phenomenon, it is necessary to design a coupler with a large difference between the main-coupling and the cross-coupling even when the system is in a misalignment state. The specific coupler design for overcoming the null power point can be dealt with in future work.

## Figures and Tables

**Table 1:** RLC Meter Measured Capacitance Components.

Parameter	$\mathrm{Copper}\;\mathrm{Plate}\;(D_{x}\;=\;10\;mm)$	$\mathrm{Metal}\;\mathrm{Mesh}\;(D_{x}\;=\;10\;mm)$
$C_{ac}$, $C_{bd}$	12.552 pF, 12.472 pF	12.275 pF, 12.213 pF
$C_{ad}$, $C_{bc}$	1.401 pF, 1.418 pF	1.282 pF, 1.316 pF
$C_{ab}$, $C_{cd}$	1.325 pF, 1.408 pF	1.237 pF, 1.221 pF
$C_{m}$	5.57 pF	5.5 pF
$C_{1}$,$\;C_{2}$	2.8 pF	2.5 pF

**Table 2:** Experimental circuit component parameters.

Parameter	Copper Plate	Metal Mesh
$L_{R}$	20 μH	20 μH
$C_{M}$	180 pF	180 pF
$L_{M}$	1.2 μH	1 μH
$R_{p1},\;R_{p2}$	10 Ω, 1.5 Ω	10 Ω, 1.2 Ω
$C_{p1},\;C_{p2}$	2 pF, 1 pF	2 pF, 0.8 pF

**Table 3:** Plate capacitance value by $D_{y}$ misalignment.

	Point 1 20 mm	Point 2 120 mm	Point 3 135 mm	Point 4 160 mm	Point 5 250 mm
$C_{ac},\;C_{bd}$	9.33 pF	1.41 pF	1.09 pF	0.766 pF	0.286 pF
$C_{bc}$	0.39 pF	4.02 pF	5.40 pF	7.65 pF	6.82 pF
$C_{ad}$	0.18 pF	0.22 pF	0.23 pF	0.24 pF	0.21 pF
$C_{Ma}$	87.05 pF	1.988pF	1.21 pF	0.587 pF	0.082 pF
$C_{Cro}$	0.070 pF	0.884 pF	1.242 pF	1.836 pF	1.432 pF
$\left|C_{Ma}-C_{Cro}\right|$	86.98 pF	1.104 pF	0.032 pF	1.249 pF	1.35 pF
$C_{m}$	4.528 pF	0.156 pF	0.006 pF	0.134 pF	0.180 pF

## Data Availability

Not applicable.
